# Bifidobacterium depletion promotes goiter via gut-thyroid axis: evidence from Mendelian randomization and experimental validation

**DOI:** 10.3389/fmicb.2025.1621167

**Published:** 2025-07-07

**Authors:** Wenyong Liao, Yang Jiang, Jiwen Zhang, Yinghao Wu, Xue Yu, Shaohong Chen, Haiyan Liu, Linlin Xiu, Gansheng Zhong

**Affiliations:** Beijing University of Chinese Medicine, Beijing, China

**Keywords:** gut microbiota, goiter, Mendelian randomization, 16S rRNA sequencing, Bifidobacterium, gut-thyroid axis

## Abstract

**Background:**

While gut microbiota dysbiosis has been associated with thyroid disorders, its causal role in goiter pathogenesis remains unclear. We aimed to investigate whether specific gut microbial taxa causally influence goiter risk through the short-chain fatty acid (SCFA)-iodine-thyroid axis.

**Methods:**

We performed Mendelian randomization (MR) analysis using gut microbiota genome-wide association study (GWAS) data (MiBioGen consortium, *n* = 18,340) and goiter GWAS data (FinnGen R10, 10,312 cases/401,869 controls). Experimental validation included: (1) establishing a propylthiouracil (PTU)-induced goiter rat model with 16S rRNA sequencing of fecal samples, (2) targeted SCFAs quantification, (3) thyroid/serum iodine measurement, (4) thyroid hormone assays, and (5) sodium-iodide symporter (NIS) protein expression analysis.

**Results:**

MR analysis identified 10 gut microbial taxa causally associated with goiter risk (all *p* < 0.05), with *Bifidobacterium bifidum* showing protective effects (OR = 0.861, 95% CI: 0.764–0.971, *p* = 0.014). In goiter rats, 16S rRNA sequencing revealed eight differentially abundant microbial taxa including significantly reduced *B. bifidum*, accompanied by: (1) impairment of two butyrate synthesis pathways, (2) decreased levels of six SCFAs (including butyrate), (3) impaired thyroid iodine uptake, (4) downregulated NIS expression, and (5) thyroid dysfunction [reduced triiodothyronine (T3), thyroxine (T4), free T3 (FT3), free T4 (FT4) with elevated thyroid-stimulating hormone (TSH)] - all measurements showing statistical significance (*p* < 0.05).

**Conclusion:**

This study provides causal evidence that Bifidobacterium depletion may contribute to goiter development through SCFA-mediated impairment of NIS-dependent iodine uptake and thyroid hormone synthesis, highlighting the association of the “gut-thyroid axis” and laying the foundation for early prevention and therapeutic intervention of goiter.

## Graphical abstract

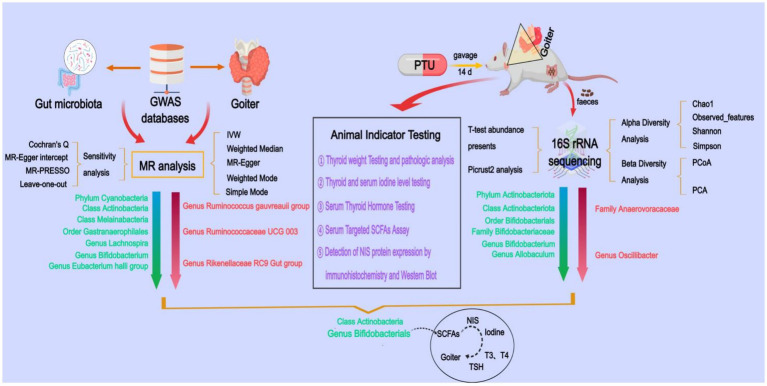

## 1 Introduction

Goiter is characterized by nodular enlargement on both sides of the anterior neck, specifically at the laryngeal nodes, resulting from hyperplasia of thyroid follicular cells accompanied by neoangiogenesis and connective tissue hyperplasia (Yildirim Simsir et al., 2020). It can manifest in various thyroid disorders, including endemic goiter, autoimmune thyroid disease, nodular goiter, thyroid cancer, thyroid granuloma, and invasive thyroid disease (Can and Rehman, 2022). A key pathological process in goiter development is the increased secretion of thyroid-stimulating hormone (TSH), which can be triggered by factors such as iodine deficiency, thyroid hormone disorders, and drug-induced effects (Hoang and Trinh, 2020). The prevalence of goiter is closely linked to the severity of iodine deficiency, with incidence rates exceeding 30% in regions with severe iodine deficiency. The widespread use of iodized salt has significantly reduced goiter incidence in such areas (Liang et al., 2017). However, the prevalence remains high in cases involving multiple thyroid disorders, such as Graves’ disease and invasive thyroid disease (Knobel, 2016; Wémeau et al., 2018; Li et al., 2021). Goiter can progressively develop into more severe thyroid conditions, including thyroid cancer, making it a persistent global public health concern.

The “gut-thyroid axis” represents a bidirectional communication pathway between the gut and the thyroid gland. This connection is rooted in the shared embryonic origin of gastric mucosal cells and thyroid follicular cells, both derived from the endoderm, as well as the anatomical link between the thyroid gland and the enteric plexus via the vagus nerve. Thyroid follicular cells, intestinal mucosa, and gastric mucosa exhibit similar functional and morphological characteristics. The gut and thyroid interact through hormones, neurotransmitters, and signaling molecules to maintain metabolic homeostasis (Lerner et al., 2017; Su et al., 2020; Huo et al., 2021; Ishaq et al., 2022; Virili et al., 2024; Zhu et al., 2024). The gut microbiota, often referred to as the “second genome” of humans, plays a pivotal role in facilitating this “gut-thyroid” communication (Cao et al., 2023). Disruptions in the gut microbiota can impair thyroid function and contribute to the development of thyroid diseases.

The human gut microbiota is a complex, dynamic, and spatially heterogeneous ecosystem comprising bacteria, fungi, archaea, and viruses. These microbial taxa interact with each other and with the human host, influencing various physiological processes (Fan and Pedersen, 2021). Notably, the bidirectional relationship between gut microbiota and thyroid function has been implicated in oncogenesis, including melanoma development. Specifically, gut microbial dysbiosis may promote carcinogenesis through its dual impact on: (1) thyroid hormone metabolism and (2) host immune responses (Ulisse et al., 2022; Luke et al., 2024; Routy et al., 2024). Emerging evidence underscores the crucial role of gut microbiota in maintaining thyroid hormone homeostasis through its regulatory effects on iodine metabolism. The gut microbiome influences thyroid function by modulating key processes in iodine handling, including intestinal absorption, metabolic conversion, and tissue storage (Knezevic et al., 2020). Specific gut microbial taxa (particularly Bifidobacterium spp.) exhibit iodine-binding properties through their metabolic activity, including the production of SCFAs (butyrate, acetate, propionate, etc.) and other bioactive compounds that regulate the function of the NIS, a pivotal transmembrane protein responsible for active iodine transport in mammalian systems. As the gatekeeper of thyroid hormone biosynthesis, NIS-mediated iodine accumulation in thyrocytes represents the rate-limiting first step in this critical endocrine pathway (Samimi and Haghpanah, 2020; Tripodi et al., 2021; Ravera et al., 2022; Sessa et al., 2025).

The gut microbiota-NIS regulatory axis constitutes a vital physiological mechanism, where microbial dysbiosis or direct NIS inhibition can impair iodine utilization, subsequently disrupting thyroid hormone synthesis and leading to systemic thyroid dysfunction. This cascade has profound pathophysiological consequences: inadequate iodine availability not only compromises normal thyroid hormone production but also triggers a compensatory pituitary response characterized by TSH hypersecretion. Chronic TSH stimulation subsequently induces thyroid follicular hyperplasia, representing a fundamental pathological mechanism in goiter development (Fröhlich and Wahl, 2019; Jiang et al., 2022).

While a clear link exists between gut microbiota and goiter, establishing causality remains challenging due to ethical constraints and the high costs associated with clinical trials. MR offers a powerful analytical approach to infer causality by using genetic variation as an instrumental variable, thereby minimizing the influence of environmental confounders and reverse causality (Sekula et al., 2016; Xu et al., 2021). Additionally, 16S rRNA sequencing has become a cornerstone of microbial research, enabling high-sensitivity, high-throughput, and widely applicable analyses in fields such as diversity studies, clinical diagnostics, and ecological research (Johnson et al., 2019; Moon et al., 2021). In this study, we employed MR to identify gut microbiota causally associated with goiter, followed by validation through animal experiments and 16S rRNA sequencing. This approach aims to pinpoint specific pathogenic or protective gut microbiota linked to goiter, providing insights into the role of gut microbiota in goiter pathogenesis and potential therapeutic interventions.

## 2 Methods

### 2.1 Two-sample Mendelian randomization analysis

**Figure 1:**
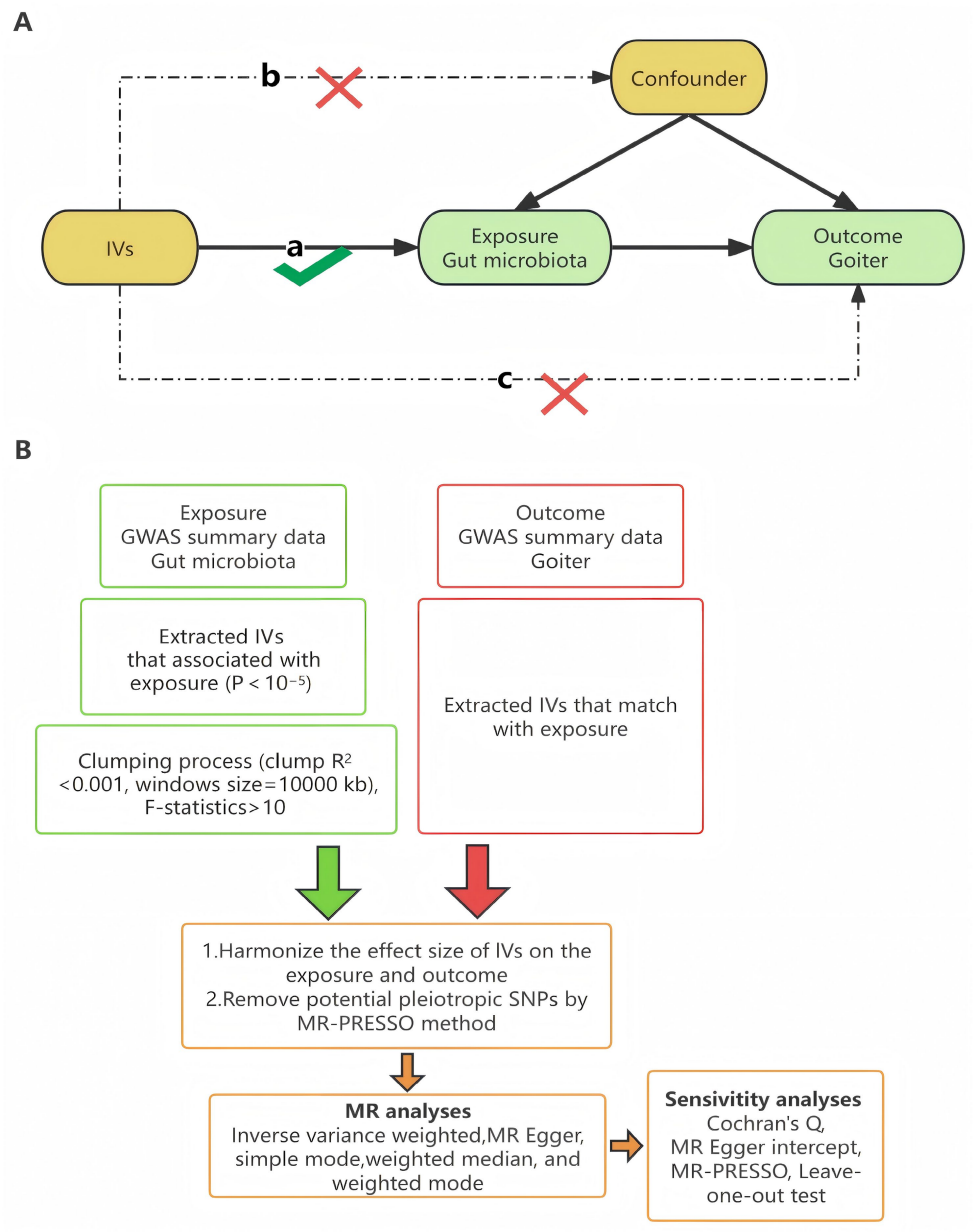
MR research hypothesis and workflow diagram. **(A)** Three major assumptions of Mendelian randomization. **(B)** Workflow Diagram. LD, Linkage Disequilibrium; SNPs, Single Nucleotide Polymorphisms; MR-PRESSO, MR Pleiotropic Residual Sum and Outliers.

This study employed a two-sample MR design, with gut microbiota as the exposure factor and goiter as the outcome factor. To ensure the validity of the MR analysis, we adhered to the three key assumptions of MR (Bowden et al., 2015): (a) The genetic variants used as instrumental variables (IVs) must be strongly associated with the exposure (gut microbiota). (b) The genetic variants must not be associated with any confounders influencing both gut microbiota and goiter. (c) The genetic variants must affect goiter exclusively through gut microbiota and not via alternative pathways (Figure 1A).

Gut microbiota-associated GWAS data were obtained from the MiBioGen International Consortium,^1^ encompassing 211 microbial taxa, including 9 phyla, 16 classes, 20 orders, 35 families (3 unknown), and 131 genera (12 unknown), with 122,110 associated single nucleotide polymorphisms (SNPs) (Kurilshikov et al., 2021). Goiter-related GWAS data were sourced from the FinnGen Consortium (R10 version, https://r10.finngen.fi/), comprising 10,312 cases and 401,869 controls, with a dataset of 19,682,705 SNPs.

The MR analysis was conducted as follows:

- Selection of IVs: SNPs associated with gut microbiota (*p*-values < 1 × 10^−5^) were selected as IVs. Linkage disequilibrium (LD) was assessed using an r < sup > 2</sup > threshold of 0.001 and a window size of 10,000 kb.
- Exclusion of Weak IVs: SNPs with F-statistics < 10, calculated using the formula F = beta^2^ (exposure) /se^2^ (exposure), were excluded to avoid weak instrument bias (Xie et al., 2023).
- Horizontal Pleiotropy Testing: The filtered IVs were merged with outcome data and tested for horizontal pleiotropy using MR-PRESSO, which identifies and removes outlier IVs.
- MR Analysis: Causal effects were estimated using five MR methods: Inverse Variance Weighting (IVW), MR-Egger, Weighted Median, Weighted Mode, and Simple Mode. The IVW method served as our principal analytical approach due to its optimal statistical properties for causal inference under valid instrumental variable assumptions.
- Sensitivity Analysis: Robustness of the results was assessed through MR-PRESSO, MR-Egger intercept tests, Cochran’s Q test, and leave-one-out analysis.

The analysis workflow is illustrated in Figure 1B. All statistical analyses were performed in R (version 4.3.3) using the TwoSampleMR (version 0.6.3) and MR-PRESSO (version 1.0) packages.

### 2.2 Establishment of the goiter rat model

Twelve Wistar rats (180–220 g) were purchased from Sibefu Biotechnology Co., Ltd. (Beijing, China) and acclimatized for 1 week at the Animal Experiment Center of Beijing University of Traditional Chinese Medicine under controlled conditions (24 ± 2°C, 50 ± 5% humidity, 12-h light/dark cycle) with free access to food and water. After acclimatization, the rats were randomly divided into control group and model group (6 rats per group). The model group was dosed with PTU (0.01 g/kg) in a volume of 10 mL/kg, and the control group was dosed with the same volume of saline. The treatment was continued for 14 days. All experimental procedures were conducted in compliance with the guidelines of the Chinese Society for Laboratory Animals and approved by the Medical Ethics Committee of Beijing University of Traditional Chinese Medicine (approval number BUCM-2024111202-4128).

### 2.3 Tissue sample, serum, and feces collection

One hour after the final gavage, fresh feces samples were collected from each rat, placed in freezing tubes, and stored at −80°C. Rats were anesthetized using isoflurane (4% for induction, 2% for maintenance), and blood was drawn from the abdominal aorta. The serum was separated and stored at −20°C. The thyroid glands were excised, photographed, weighed, and recorded. The left thyroid tissues were frozen at −80°C, while the right thyroid glands were fixed in 4% paraformaldehyde for further analysis.

### 2.4 DNA extraction and 16S rRNA amplification

Total genome DNA from feces was extracted using CTAB method. DNA concentration and purity was monitored on 1% agarose gels. According to the concentration, DNA was diluted to 1 ng/μL using sterile water. 16S rRNA genes of distinct regions (16SV4, 16SV3- V4, 16SV4- V5, 16SV5- V7) were amplified used specific primer (e.g., 16SV4: 515F- 806R, 16SV3-V4: 341F- 806R, 16SV4- V5: 515F- 907R, 16SV5- V7: 799F- 1193R) with barcodes. All PCR reactions were carried out with 15 μL of Phusion High - Fidelity PCR Master Mix; 0.2 μM of forward and reverse primers, and about 10 ng template DNA. Thermal cycling consisted of initial denaturation at 98°C for 1 min, followed by 30 cycles of denaturation at 98°C for 10 s, annealing at 50°C for 30 s, and elongation at 72°C for 30 s and 72°C for 5 min.

### 2.5 16S rRNA amplicon library preparation and sequencing

Sequencing libraries were generated using TruSeq® DNA PCR- Free Sample Preparation Kit (Illumina, USA) following manufacturer’s recommendations and index codes were added. The library quality was assessed on the Qubit@ 2.0 Fluorometer (Thermo Scientific) and Agilent Bioanalyzer 2,100 system. At last, the library was sequenced on an Illumina NovaSeq platform and 250 bp paired-end reads were generated.

### 2.6 Bioinformatics analysis of 16S rRNA sequencing

Paired-end reads were merged using FLASH (V1.2.7, http://ccb.jhu.edu/software/FLASH/) to generate Raw Tags. The Raw Tags were then rigorously filtered using the fastp software (V 0.23.1) to obtain high-quality Clean Tags. To ensure data accuracy, chimeric sequences were identified and removed by comparing the Tags with the Silva database^2^ (Edgar et al., 2011). The resulting Effective Tags were further processed using the DADA2 module in QIIME2 software for noise reduction, yielding Amplicon Sequence Variants (ASVs), which were subsequently annotated to taxa level (Wang et al., 2021).

We assessed alpha diversity using the Chao1, observed_features, Shannon, and Simpson indices to evaluate taxa richness and evenness. Beta diversity was visualized in R software (version 4.0.3) through Principal Coordinates Analysis (PCoA) and Principal Component Analysis (PCA) to examine community structure differences. Based on taxa annotation results, the top 10 most abundant taxa at each taxonomic level (Phylum, Class, Order, Family, Genus) were selected for each subgroup. Microbial community composition was visualized using relative abundance distribution histograms generated with Perl’s SVG function. Differential abundance analysis of microbial taxa between groups was performed using Student’s *t-*test *p* < 0.05 considered significant. Functional profiling was conducted via PICRUSt2 to predict KEGG metabolic pathways, with pathway abundance differences assessed using t-tests.

### 2.7 Serum-targeted SCFAs assay

The serum was homogenized in 80% methanol and centrifuged at 12,000 rpm for 10 min to precipitate proteins. The supernatant was derivatized with 150 μL of derivatization reagent at 40°C for 40 min, followed by dilution with 80% methanol. Subsequently, 95 μL of the supernatant was mixed with 5 μL of internal standard solution and analyzed by LC–MS/MS. A calibration curve was constructed by plotting the ratio of standard concentration to internal standard concentration (x-axis) against the ratio of their peak areas (y-axis). Linearity was confirmed with a correlation coefficient (r) > 0.99 for all metabolites. The limit of quantification (LOQ) was determined at a signal-to-noise ratio (S/N) of 10, based on the lowest detectable concentration in the blank matrix. All of the 11 SCFAs standards were obtained from ZZ Standards Co., LTD. (Shanghai, China). Methanol (Optima LC–MS), acetonitrile (Optima LC–MS), ammonium acetate, isopropanol (Optima LC–MS) were purchased from Thermo-Fisher Scientific (FairLawn, NJ, USA). Ultrapure water was purchased from Millipore (MA, USA). A ultra-high performance liquid chromatography coupled to tandem mass spectrometry (UHPLC–MS/MS) system (Vanquish™ Flex UHPLC-TSQ Altis™, Thermo Scientific Corp., Germany).

### 2.8 Thyroid and serum iodine content measurement, enzyme-linked immunosorbent assay (ELISA)

Thyroid and serum iodine content was quantified using an atomic fluorescence photometer. Serum levels of T3, T4, TSH, FT3, and FT4 were measured using ELISA kits (Huaying Biotechnology Research Institute, Beijing, China) according to the manufacturer’s instructions. Absorbance was read at 450 nm using a microplate reader.

### 2.9 Histopathological analysis [hematoxylin and eosin (H&E) staining]

The right thyroid glands were fixed, paraffin-embedded, and sectioned at 4-μm thickness. Tissue sections were stained with H&E following standard protocols, then examined under a upright light microscope (Nikon Eclipse E100, Nikon Corporation., Japan, 400 × magnification). Representative images were captured for morphological evaluation, focusing on follicular structure, nuclear density, colloid retention, and epithelial cell changes.

### 2.10 Immunohistochemistry (IHC) analysis of thyroid NIS protein expression

Paraffin-embedded thyroid tissue sections were deparaffinized, subjected to antigen retrieval, and blocked with 5% goat serum (room temperature, 30 min). The sections were then incubated with a rabbit polyclonal anti-NIS primary antibody (Proteintech, #24324-1-AP, 1:100 dilution) at 37°C for 1 h, followed by three washes with phosphate-buffered saline (PBS; 5 min each). Subsequently, the sections were treated with horseradish peroxidase (HRP)-conjugated secondary antibody (room temperature, 45 min) and visualized using a 3,3′-diaminobenzidine (DAB) substrate kit. Images were captured under an upright light microscope (Nikon Eclipse E100, Nikon Corporation., Japan, 400 × magnification), and positively colored cells were counted in multiple random fields of view.

### 2.11 Western blot (WB) analysis of thyroid NIS protein expression

Frozen thyroid tissues were homogenized in RIPA lysis buffer supplemented with protease and phosphatase inhibitors (100:1:1 ratio). Total protein was extracted by centrifugation (12,000 × g, 15 min, 4°C) and quantified using a bicinchoninic acid (BCA) assay kit (KeyGEN BioTECH, China). Equal amounts of protein (40 μg per lane) were separated by 10% SDS-PAGE and transferred onto polyvinylidene difluoride (PVDF) membranes. After blocking with 5% non-fat milk for 2 h at room temperature, the membranes were probed with the anti-NIS primary antibody (1:1000 dilution) and corresponding HRP-conjugated secondary antibody (Proteintech, #SA00001-1, 1:5000 dilution), each incubated for 1.5 h at room temperature. Protein bands were detected using an enhanced chemiluminescence (ECL) kit and quantified using ImageJ software (NIH, USA), with *β*-actin (Proteintech, #20536-1-AP, 1:10,000) serving as the loading control.

### 2.12 Statistical analysis

Statistical analyses were performed using SPSS 27.0. Data are presented as mean ± standard deviation. Differences between groups were analyzed using Student’s t-test, with a *p*-value < 0.05 considered statistically significant. For alpha diversity analysis, the Wilcoxon rank-sum test was applied, with a *p*-value < 0.05 indicating statistical significance. Beta diversity analysis was visualized using PCoA, with Unweighted UniFrac distances calculated to assess community structure differences between groups.

## 3 Results

### 3.1 Results of Mendelian randomization

#### 3.1.1 Overall effect of gut flora on goiter

Our analysis began with data from the MiBioGen consortium, where we identified 13,671 SNPs associated with gut microbiota composition at a significance threshold of *p* < 1 × 10^−5^ (Supplementary Table S1). These SNPs corresponded to 196 distinct bacterial traits across multiple taxonomic levels: 119 genera, 32 families, 20 orders, 16 classes, 9 phyla.

After a series of quality control steps, we retained 2,428 high-quality SNPs for final analysis. All instrumental variables demonstrated strong association signals (*F*-statistic > 10). This stringent filtering ensures the robustness of our Mendelian randomization results.

**Figure 2:**
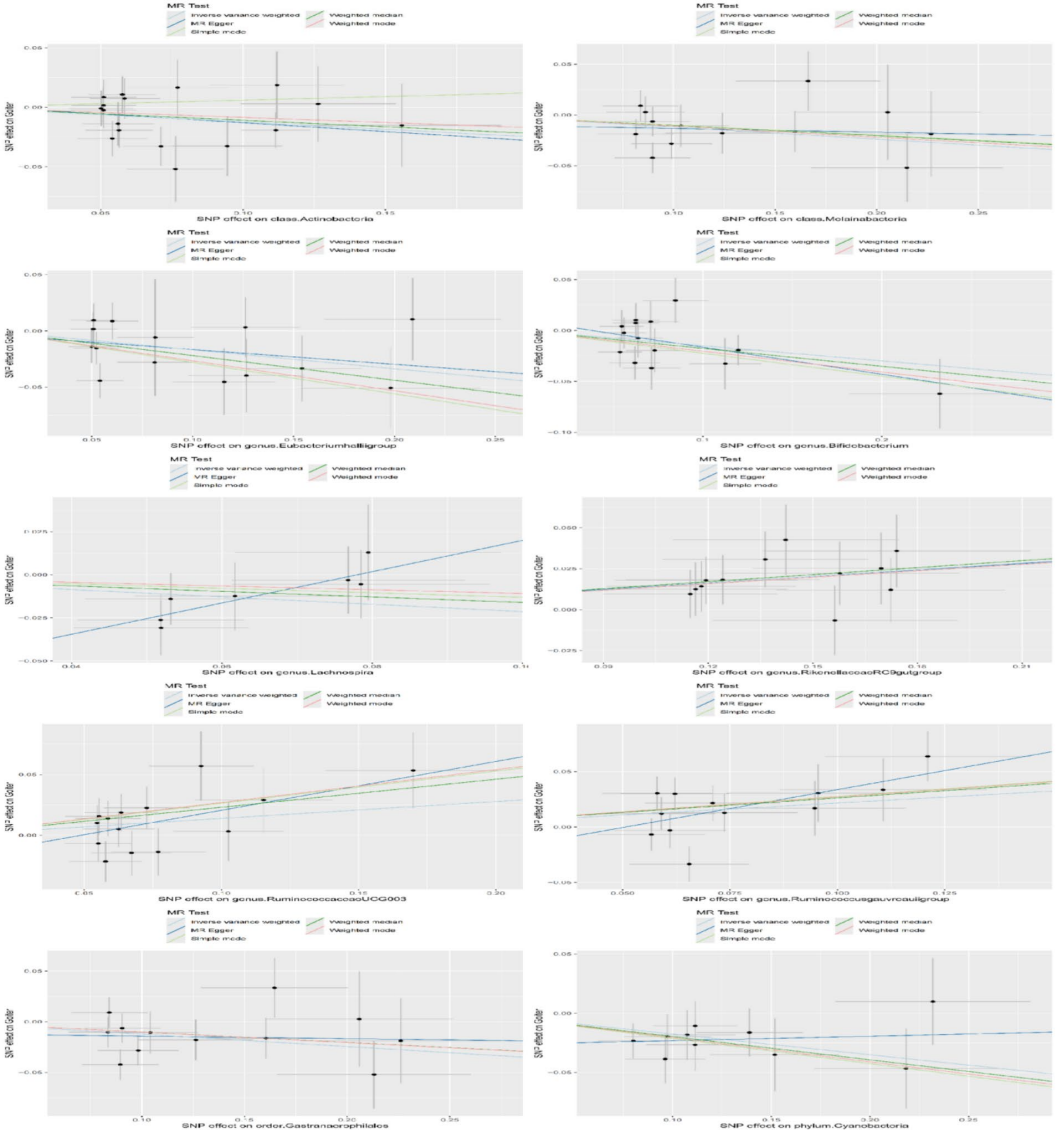
Scatterplot of the causal effects of gut microbiota on goiter risk. This scatterplot evaluates the causal effects of gut microbiota on goiter risk using MR. Each dot represents a SNP from gut microbiota GWAS data. The x-axis shows SNP effects on microbiota abundance, while the y-axis shows their effects on goiter risk. Regression lines (colored by MR method: IVW [light blue], MR-Egger [dark blue], Simple Mode [light green], Weighted Median [dark green], Weighted Mode [red]) depict causality. Positive slopes indicate risk effects; negative slopes suggest protection. Gray crosses denote standard errors.

According to the IVW analysis, genetic predictions of the genus *Ruminococcus gauvreauii* group (OR = 1.241, 95% CI: 1.045–1.474, *p* = 0.014), genus Rikenellaceae RC9 gut group (OR = 1.145, 95% CI: 1.066–1.231, *p* < 0.001), and genus Ruminococcaceae UCG 003 (OR = 1.149, 95% CI: 1.010–1.308, *p* = 0.035) were positively associated with an increased risk of goiter. Conversely, the phylum Cyanobacteria (OR = 0.839, 95% CI: 0.751–0.938, *p* = 0.002), class Actinobacteria (OR = 0.883, 95% CI: 0.784–0.996, *p* = 0.042), class Melainabacteria (OR = 0.888, 95% CI: 0.808–0.976, *p* = 0.014), order Gastranaerophilales (OR = 0.885, 95% CI: 0.803–0.976, *p* = 0.014), genus Lachnospira (OR = 0.808, 95% CI: 0.653–0.999, *p* = 0.049), genus *Eubacterium hallii* group (OR = 0.845, 95% CI: 0.743–0.962, *p* = 0.011), and genus Bifidobacterium (OR = 0.861, 95% CI: 0.764–0.971, *p* = 0.014) were positively associated with a reduced risk of goiter (Table 1). Scatter plots displaying the causal effect of the gut microbiota on goiter can be found in Figure 2.

**Table 1:** MR results of the causal effect of gut microbiota on goiter risk.

Exposure	Method	Nsnp	B	SE	*p*-value	OR (95% CI)
Genus Rikenellaceae RC9 gut group	MR Egger	12	0.131	0.236	0.591	1.140 (0.718–1.811)
	Weighted median	12	0.143	0.048	0.003	1.153 (1.050–1.266)
	Inverse variance weighted	12	0.136	0.037	<0.001	1.145 (1.066–1.231)
	Simple mode	12	0.132	0.076	0.110	1.141 (0.983–1.324)
	Weighted mode	12	0.132	0.080	0.128	1.141 (0.975–1.334)
Genus Bifidobacterium	MR Egger	14	−0.265	0.161	0.126	0.767 (0.559–1.052)
	Weighted median	14	−0.176	0.087	0.044	0.839 (0.707–0.996)
	Inverse variance weighted	14	−0.149	0.061	0.014	0.861 (0.764–0.971)
	Simple mode	14	−0.224	0.155	0.173	0.799 (0.590–1.084)
	Weighted mode	14	−0.204	0.104	0.072	0.816 (0.665–1.001)
Genus *Eubacterium hallii* group	MR Egger	14	−0.131	0.142	0.374	0.877 (0.664–1.159)
	Weighted median	14	−0.219	0.090	0.015	0.803 (0.673–0.958)
	Inverse variance weighted	14	−0.168	0.066	0.011	0.845 (0.743–0.962)
	Simple mode	14	−0.28	0.157	0.098	0.756 (0.556–1.028)
	Weighted mode	14	−0.266	0.151	0.101	0.767 (0.570–1.030)
Order Gastranaerophilales	MR Egger	12	−0.025	0.163	0.882	0.976 (0.708–1.344)
	Weighted median	12	−0.102	0.068	0.136	0.903 (0.790–1.033)
	Inverse variance weighted	12	−0.122	0.050	0.014	0.885 (0.803–0.976)
	Simple mode	12	−0.102	0.102	0.335	0.903 (0.740–1.101)
	Weighted mode	12	−0.102	0.099	0.322	0.903 (0.744–1.095)
Phylum Cyanobacteria	MR Egger	10	0.037	0.183	0.843	1.038 (0.726–1.485)
	Weighted median	10	−0.197	0.074	0.008	0.821 (0.710–0.950)
	Inverse variance weighted	10	−0.175	0.056	0.002	0.839 (0.751–0.938)
	Simple mode	10	−0.213	0.120	0.109	0.808 (0.639–1.022)
	Weighted mode	10	−0.205	0.108	0.090	0.814 (0.659–1.007)
genus *Ruminococcus gauvreauii* group	MR Egger	12	0.684	0.351	0.080	1.982 (0.997–3.943)
	Weighted median	12	0.263	0.096	0.006	1.300 (1.077–1.569)
	Inverse variance weighted	12	0.216	0.088	0.014	1.241 (1.045–1.474)
	Simple mode	12	0.275	0.163	0.120	1.317 (0.956–1.814)
	Weighted mode	12	0.272	0.174	0.146	1.312 (0.934–1.844)
Class Melainabacteria	MR Egger	13	−0.037	0.159	0.822	0.964 (0.706–1.316)
	Weighted median	13	−0.102	0.066	0.124	0.903 (0.793–1.028)
	Inverse variance weighted	13	−0.119	0.048	0.014	0.888 (0.808–0.976)
	Simple mode	13	−0.100	0.105	0.360	0.905 (0.737–1.111)
	Weighted mode	13	−0.109	0.106	0.324	0.896 (0.728–1.104)
Genus Ruminococcaceae UCG 003	MR Egger	14	0.402	0.222	0.095	1.495 (0.967–2.309)
	Weighted median	14	0.231	0.091	0.011	1.260 (1.055–1.504)
	Inverse variance weighted	14	0.139	0.066	0.035	1.149 (1.010–1.308)
	Simple mode	14	0.264	0.161	0.124	1.303 (0.951–1.785)
	Weighted mode	14	0.270	0.148	0.092	1.311 (0.980–1.753)
Genus Lachnospira	MR Egger	7	0.512	0.307	0.133	1.670 (0.915–3.048)
	Weighted median	7	−0.161	0.150	0.285	0.851 (0.634–1.143)
	Inverse variance weighted	7	−0.214	0.108	0.049	0.808 (0.653–0.999)
	Simple mode	7	−0.131	0.211	0.557	0.877 (0.581–1.326)
	Weighted mode	7	−0.109	0.194	0.595	0.897 (0.614–1.311)
Class Actinobacteria	MR Egger	17	−0.149	0.182	0.428	0.862 (0.603–1.232)
	Weighted median	17	−0.109	0.088	0.214	0.897 (0.755–1.065)
	Inverse variance weighted	17	−0.124	0.061	0.042	0.884 (0.784–0.996)
	Simple mode	17	0.060	0.147	0.686	1.062 (0.797–1.416)
	Weighted mode	17	−0.085	0.121	0.491	0.918 (0.725–1.164)

Nsnp, number of SNPs; B, effect size estimate (slope value); SE, standard error; OR, odds ratio; 95% CI, 95% confidence interval.

#### 3.1.2 Sensitivity analysis

**Figure 3:**
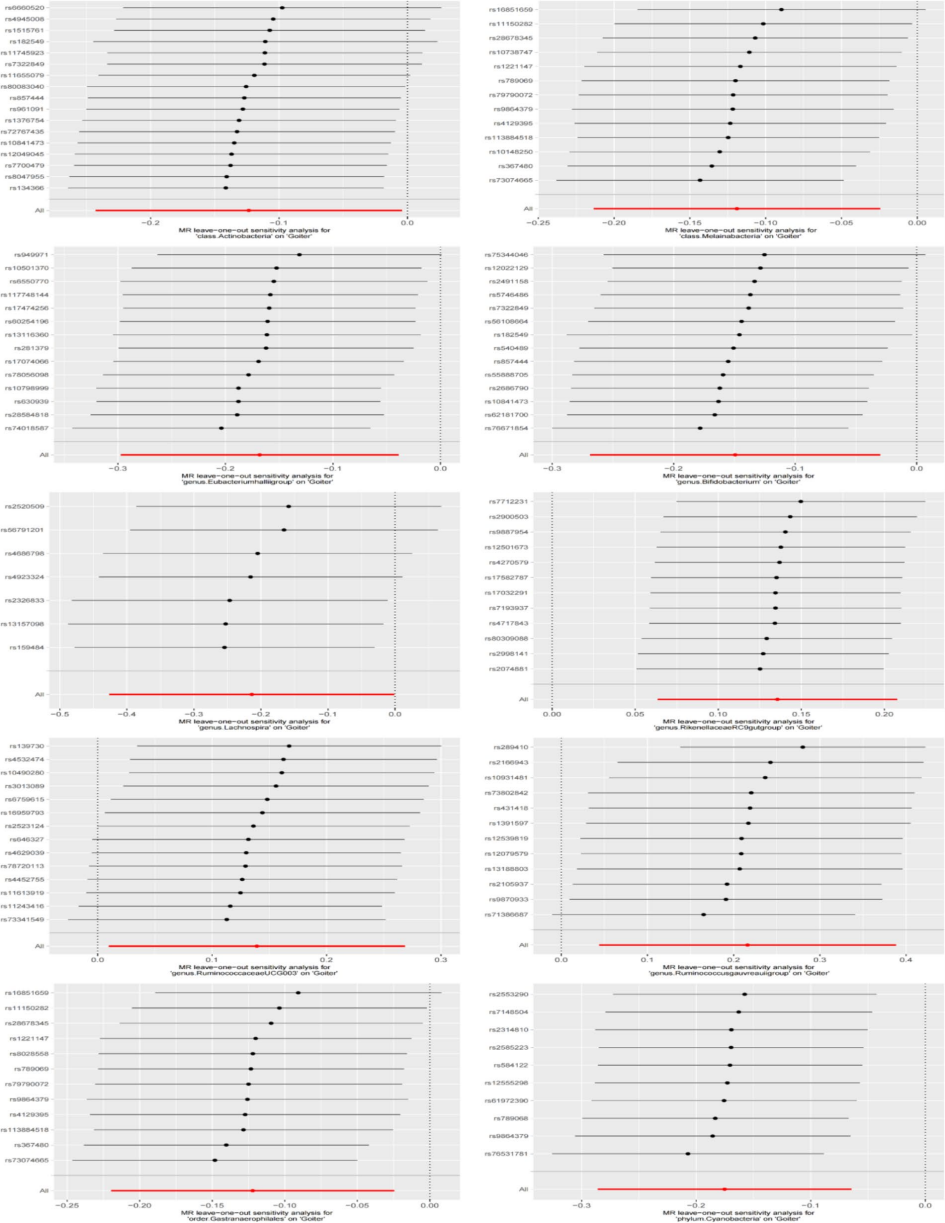
Diagram of the leave-one-out method of analysis. The leave-one-out method was used to assess the sensitivity of specific gut microbiota to the causal effects of goiter. Dots represent MR estimates for the gut microbiota on goiter using the IVW fixed-effect method, with each SNP omitted in turn. Error lines indicate the 95% confidence intervals using the IVW method.

We conducted a series of sensitivity analyses to evaluate the heterogeneity and horizontal pleiotropy of the selected instrumental variables (Table 2). Using Cochran’s Q test, we found no significant heterogeneity (*p* > 0.05). Both the MR-Egger intercept test and MR-PRESSO global test confirmed the absence of horizontal pleiotropy (*p* > 0.05). Additionally, the leave-one-out test indicated that no SNPs had influential outlier effects (Figure 3).

**Table 2:** Sensitivity analysis of the results of MR analysis of gut microbiota on goiter risk.

Outcome	Exposure	Cochran Q statistic	Cochran Q *p*-value	MR-Egger Intercept	Intercept *p*-value	MR-PRESSOGlobal test *p*-value
Goiter	Class Actinobacteria	14.565	0.557	0.002	0.886	0.594
	Class Melainabacteria	12.850	0.380	−0.010	0.596	0.434
	Genus *Eubacterium hallii* group	13.155	0.436	−0.003	0.771	0.474
	Genus Bifidobacterium	13.214	0.431	0.010	0.451	0.500
	Genus Lachnospira	4.588	0.598	−0.071	0.111	0.615
	Genus Rikenellaceae RC9 gut group	4.321	0.960	0.001	0.985	0.966
	Genus Ruminococcaceae UCG 003	12.577	0.481	−0.020	0.239	0.521
	Genus *Ruminococcus gauvreauii* group	17.579	0.092	−0.035	0.199	0.092
	Order Gastranaerophilales	11.722	0.385	−0.012	0.545	0.403
	Phylum Cyanobacteria	4.077	0.906	−0.027	0.256	0.914

### 3.2 *In vivo* experimental results in animal models

#### 3.2.1 Validation of the rat goiter model

In this experiment, a rat goiter model was successfully established by continuous gavage of PTU, a drug used to treat hyperthyroidism. PTU inhibits thyroid hormone synthesis by blocking the peroxidase system in the thyroid gland, thereby preventing the iodination of tyrosine and the condensation of iodinated tyrosine. This inhibition ultimately leads to compensatory enlargement of the thyroid gland. Consistent with our previous studies (Li C. et al., 2022; Li et al., 2023; Wu et al., 2025), PTU administration in the current experiment effectively induced goiter in rats.

**Figure 4:**
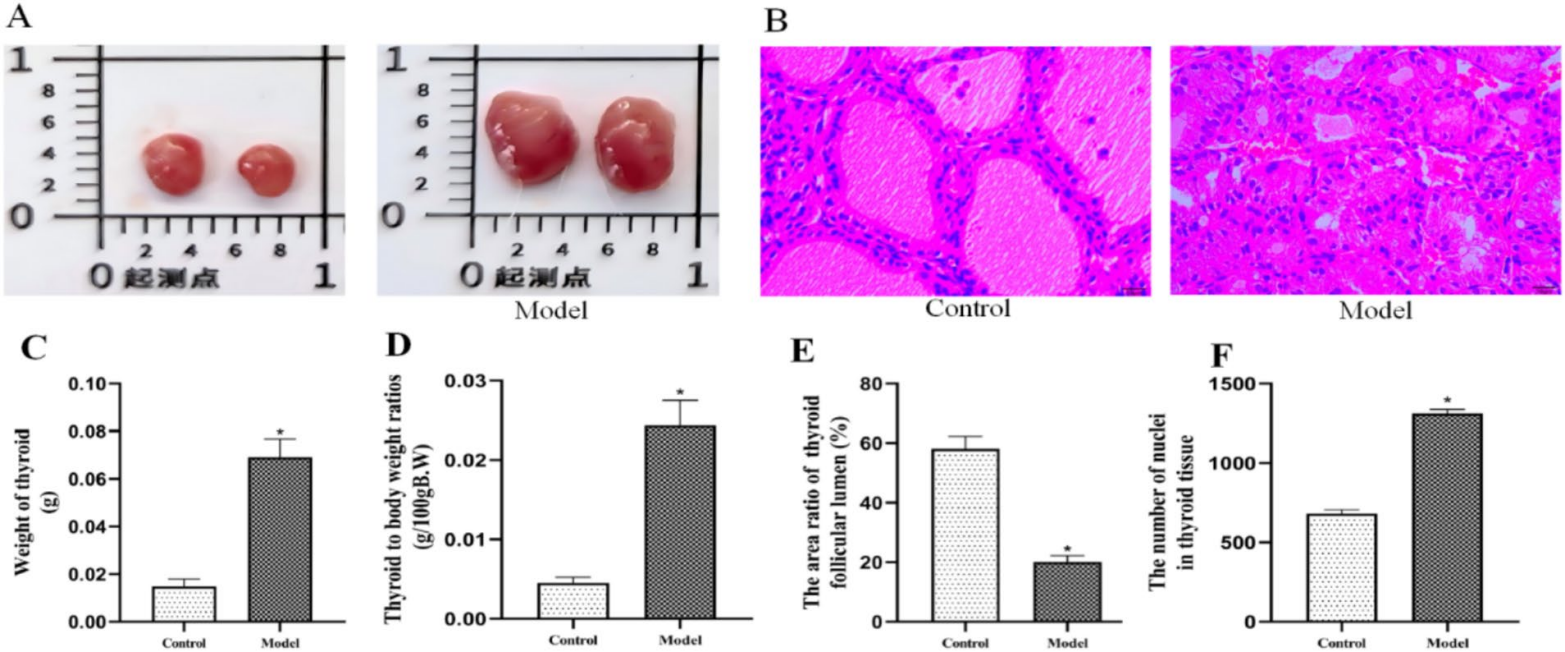
PTU-induced goiter model validation. **(A)** Thyrograms of each group, **(B)** thyroid pathograms of each group (H&E staining, all scale bars, 50 μm), **(C)** thyroid weight, **(D)** thyroid to body weight ratios (thyroid coefficients), **(E)** follicular lumen area of thyroid gland, **(F)** the number of nuclei in thyroid tissue. *n* = 6, *p < 0.05 compared with the control group.

Two weeks after PTU treatment, thyroid weight and thyroid coefficient were significantly higher in the model group than in the control group (Figures 4A,C,D; *p* < 0.05). Histopathological analysis revealed distinct differences between the groups (Figure 4B). In the control group, thyroid follicles exhibited a round or oval shape, with lumens filled with colloid. The follicular epithelial cells were uniformly cuboidal and neatly arranged around the basement membrane. In contrast, the model group displayed several pathological changes, including irregularly shaped follicles, a reduction in thyroid follicle lumen area (Figure 4E; *p* < 0.05), decreased colloid content, increased nuclear density (Figure 4F; *p* < 0.05), and enlarged follicular epithelial cells.

#### 3.2.2 Differences in gut microbial diversity

**Figure 5:**
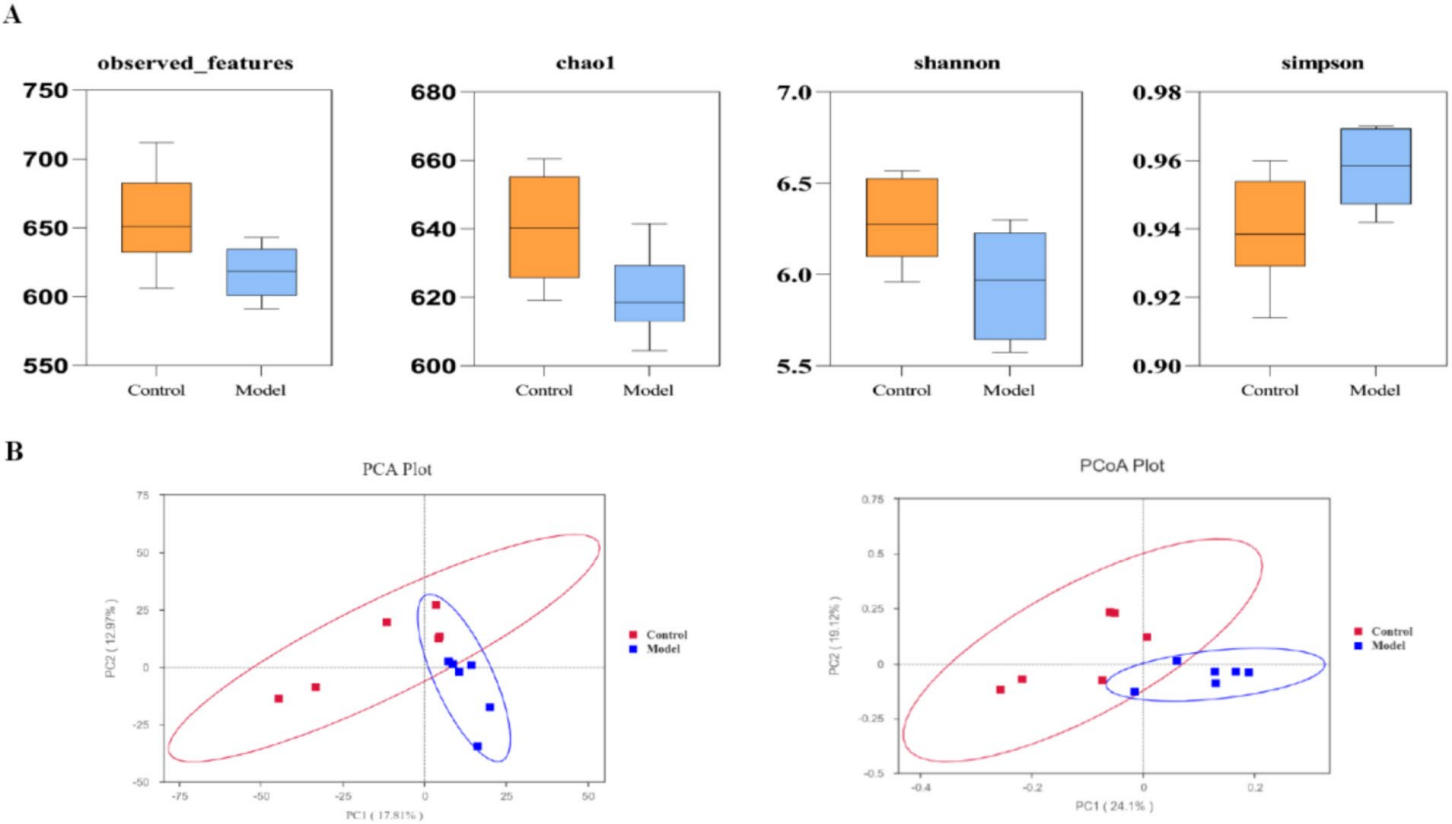
Comparison of gut microbial diversity between the control group and the model group. **(A)** Results of the *α*-diversity analysis. The Chao1, observed_features, and Shannon indices reflect taxa richness and diversity, with higher values indicating greater richness and diversity. The Simpson index, on the other hand, ranges from 0 to 1, with values closer to 1 indicating lower taxa richness and diversity. **(B)** Results of the *β*-diversity analysis. Samples with more similar microbial components and community composition are positioned closer together in the graph, while those with greater differences are positioned farther apart.

Alpha diversity analysis revealed significantly reduced microbial richness and evenness in the model group compared to controls, as evidenced by decreased Chao1 (*p* = 0.037), observed features (*p* = 0.043), Shannon (*p* = 0.048), and Simpson indices (*p* = 0.044) (Figure 5A). Beta diversity assessment through principal coordinates analysis (PCoA) and principal component analysis (PCA) demonstrated distinct clustering patterns between groups, confirming substantial alterations in community structure following goiter induction despite partial overlap (Figure 5B).

These findings suggest that the establishment of the rat goiter model led to a reduction in gut microbial diversity and significant alterations in community structure.

#### 3.2.3 Gut microbial taxa differences

**Figure 6:**
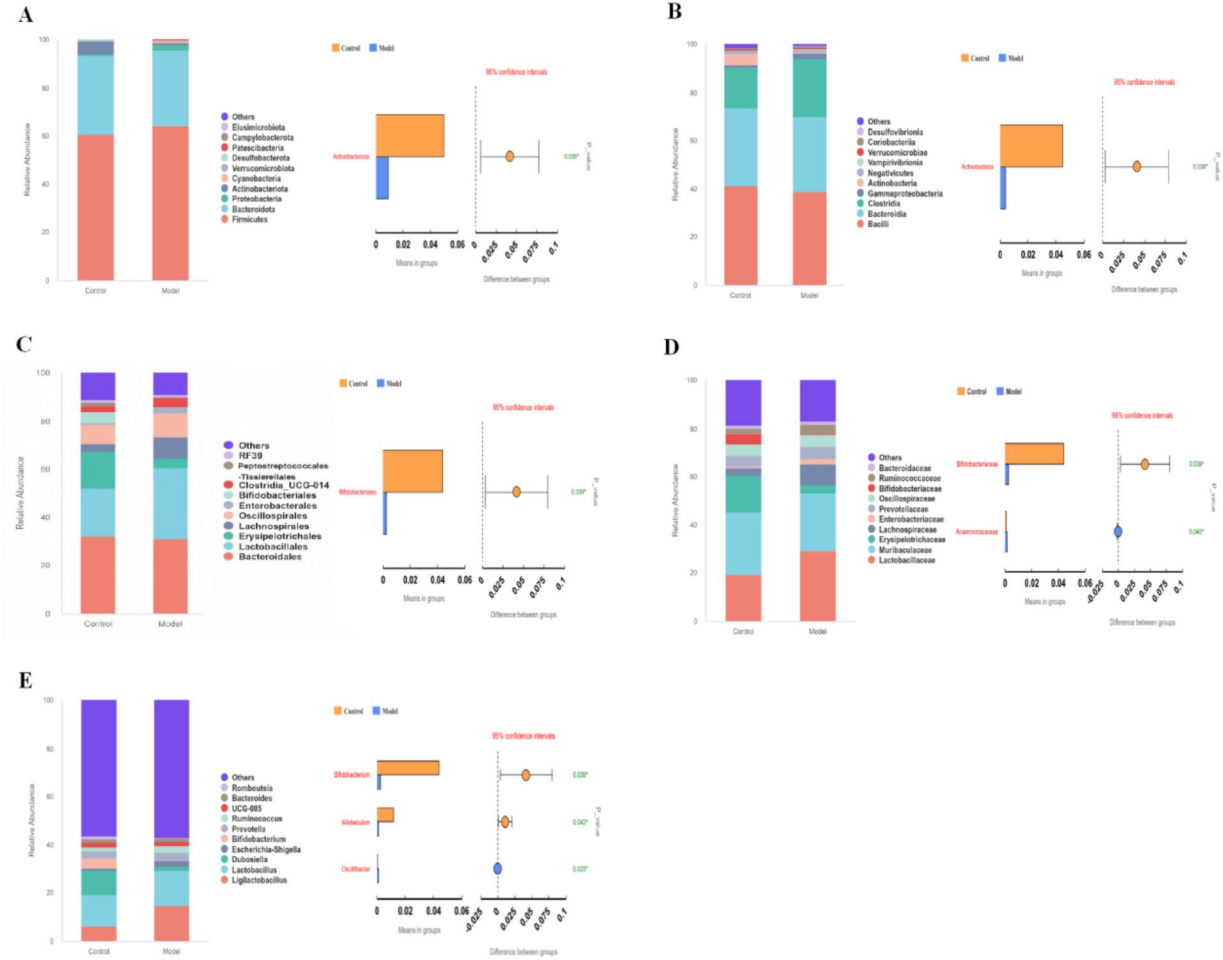
Gut microbiota abundance and divergent taxa. **(A–E)** Corresponds to the forest map of the top ten ranked gut microbial abundance and statistically different taxa at the phylum, order, family, and genus classification levels, respectively.

Based on taxa annotation, the top 10 most abundant taxa at each taxonomic level (Phylum, Class, Order, Family, Genus) were selected for further analysis. Relative abundance bar plots were generated, and statistically significant differences between groups were identified using the T-test (Figure 6).

The results showed that at the phylum level, Actinobacteriota abundance was significantly reduced in the model group compared to the control group (*p* = 0.030); at the class level, Actinobacteria abundance was significantly reduced in the model group (*p* = 0.038); at the order level, Bifidobacterials abundance was significantly reduced (*p* = 0.039); at the family level, Bifidobacteriaceae abundance was significantly reduced (*p* = 0.039) in the model group, Anaerovoracaceae abundance significantly increased (*p* = 0.045); and at the genus level, Bifidobacterium and Allobaculum abundance was significantly reduced in the model group (*p* = 0.039, *p* = 0.043), Oscillibacter abundance significantly increased (*p* = 0.022). By integrating these results with the MR findings, we identified Actinobacteria (class) and Bifidobacterium (genus) as causally associated with goiter. These microbial taxa may serve as key strains for goiter treatment.

#### 3.2.4 Prediction of microbial metabolic pathways (PICRUSt2 analysis)

**Figure 7:**
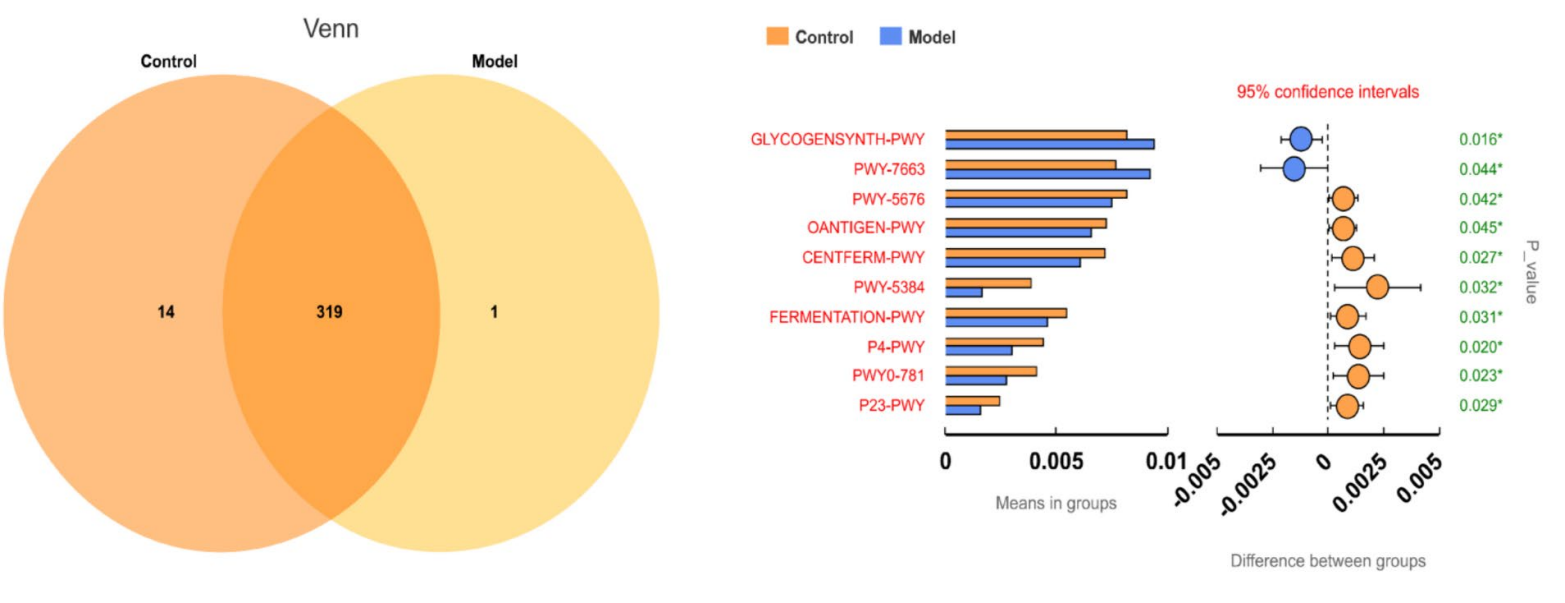
Microbial metabolic pathways. GLYCOGENSYNTH-PWY for glycogen biosynthesis I, PWY-7663 for gondoate biosynthesis (anaerobic), PWY-5676 for acetyl-CoA fermentation to butyrate II, OANTIGEN-PWY for O-antigen building blocks biosynthesis (*E. coli*), CENTFERM-PWY for pyruvate fermentation to butyrate, PWY-5384 for sucrose degradation IV (sucrose phosphorylase), FERMENTATION-PWY for mixed acid fermentation, P4-PWY for superpathway of L-lysine, L-threonine and L-methionine biosynthesis I, PWY0-781 for aspartate superpathway, P23-PWY for reductive TCA cycle I.

Comparative analysis of 16S rRNA data predicted 334 KEGG metabolic pathways, with 319 pathways shared between control and goiter groups (Figure 7). Among the top 10 differentially abundant pathways (*p* < 0.05), two SCFAs (butyrate) synthesis pathways showed significant downregulation in goiter rats, including acetyl-CoA fermentation to butyrate II (*p* = 0.042) and pyruvate fermentation to butyrate (*p* = 0.027). These reductions correlated with the observed depletion of butyrate-producing Bifidobacterium and Actinobacteria in the goiter model. Complete pathway data are provided in Supplementary Table S2.

#### 3.2.5 Targeted analysis of SCFAs

**Figure 8:**
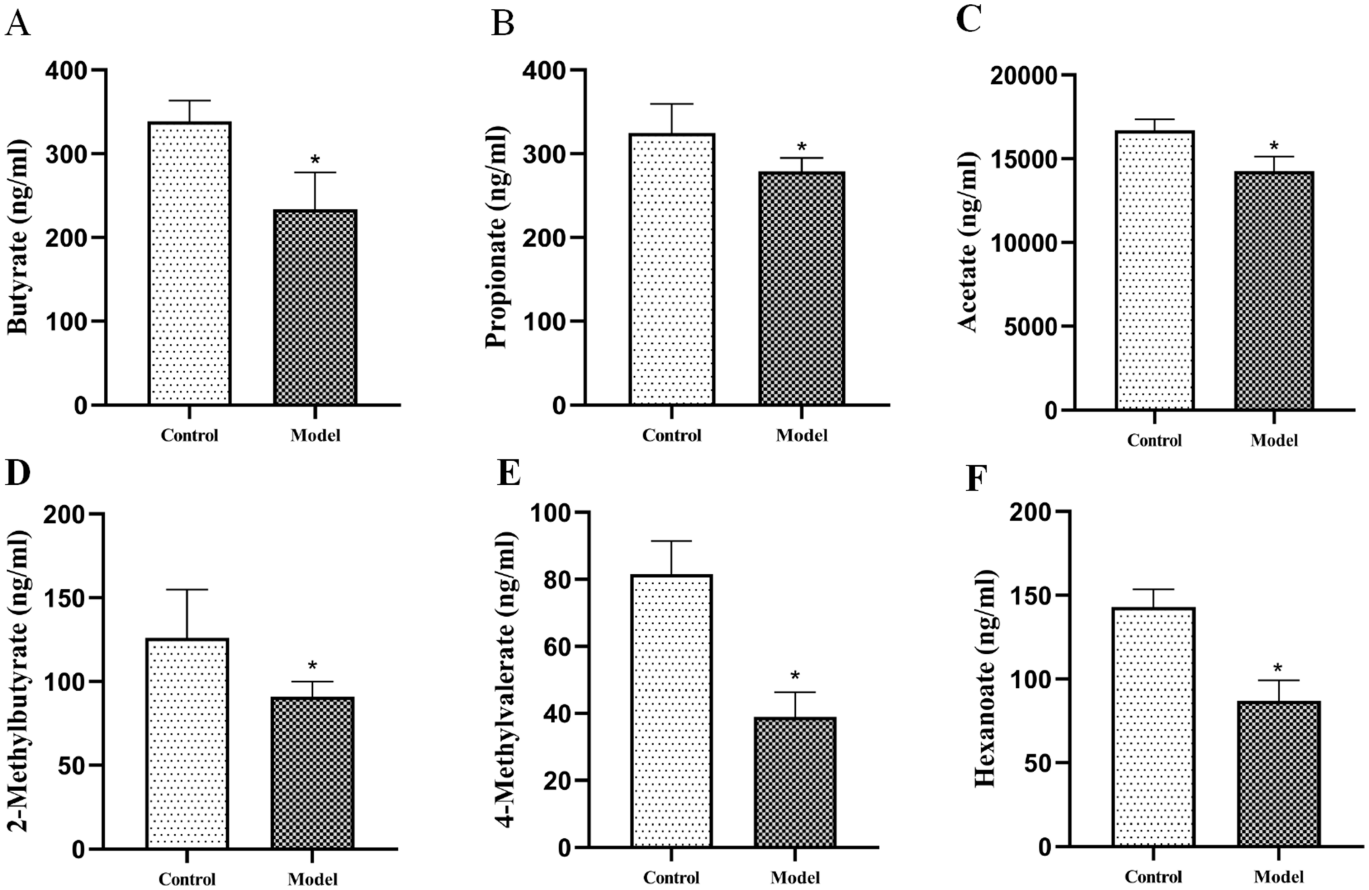
Decreased serum levels of six SCFAs in goiter model. **(A)** Butyrate, **(B)** Propionate, **(C)** Acetate, **(D)** 2-Methylbutyrate, **(E)** 4-Methylvalerate, **(F)** Hexanoate. *n* = 6, *p < 0.05 compared with the control group.

Our targeted analysis of 11 SCFAs in rat serum revealed significant reductions in six specific SCFAs in the model group compared with controls (Figures 8A–F; *p* < 0.05), including Butyrate, Propionate, Acetate, 2-Methylbutyrate, 4-Methylvalerate, and Hexanoate. These results suggest that dysregulation of specific SCFAs may contribute to goiter pathogenesis. Integrating MR and 16S rRNA sequencing findings, we propose that depletion of Actinobacteria, particularly Bifidobacterium, may impair SCFA biosynthesis pathways, ultimately reducing SCFAs production.

#### 3.2.6 Quantification of thyroid NIS protein expression

**Figure 9:**
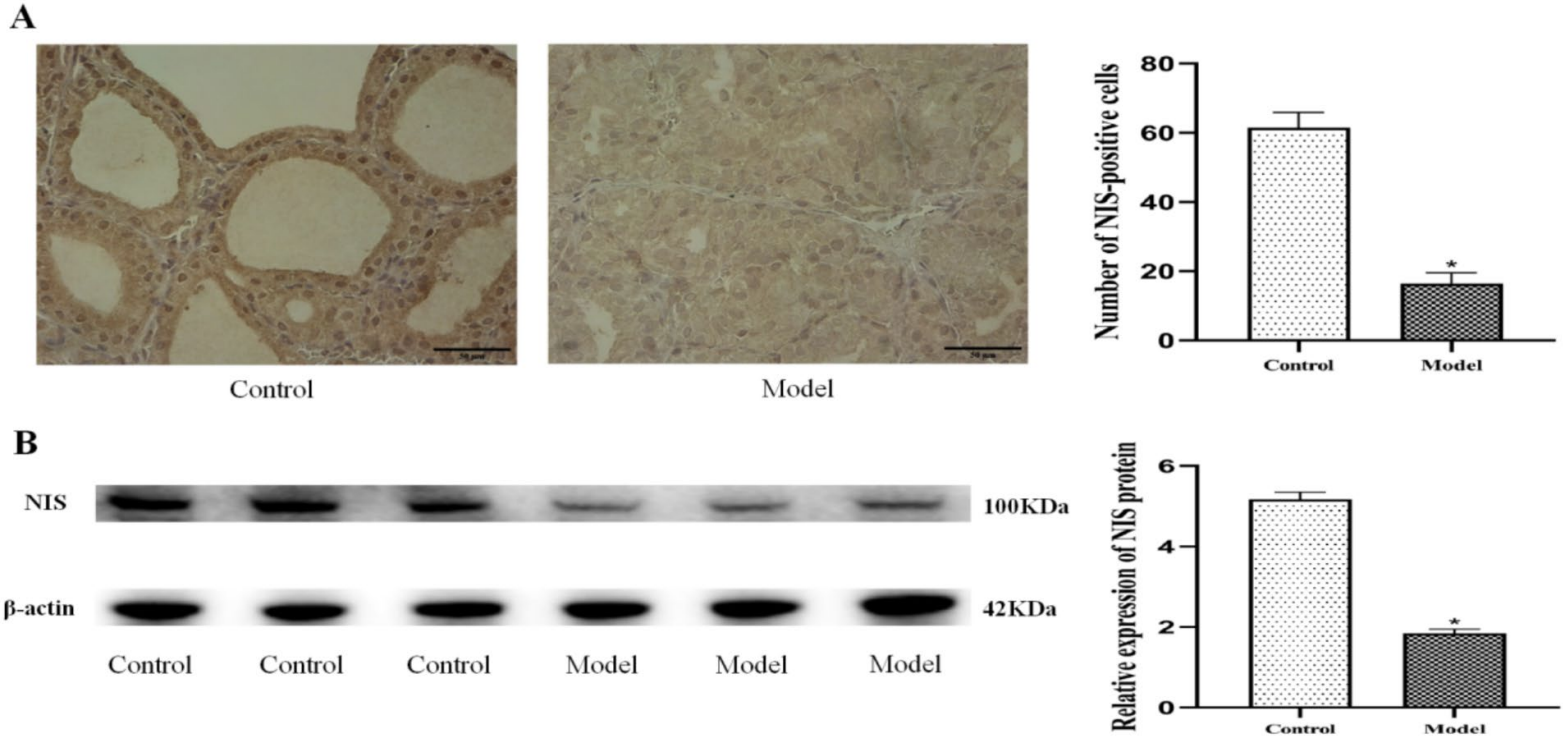
Downregulation of thyroid NIS protein expression in goiter model rats. **(A)** Representative immunohistochemical images and quantitative analysis of NIS-positive cells, all scale bars, 50 μm. **(B)** Western blot analysis and relative protein expression levels. *n* = 6, *p < 0.05 compared with the control group.

Quantitative analysis of thyroid NIS protein expression by both IHC and WB revealed significant downregulation in the model group. Immunohistochemical evaluation demonstrated a marked reduction in NIS-positive cells (Figure 9A; *p* < 0.05), while Western blot analysis confirmed decreased NIS protein expression levels compared to controls (Figure 9B; *p* < 0.05). These findings suggest that NIS dysregulation, potentially mediated by SCFA deficiency, may contribute significantly to goiter pathogenesis.

#### 3.2.7 Thyroid iodine content and serum iodine/thyroid hormone levels

**Figure 10:**
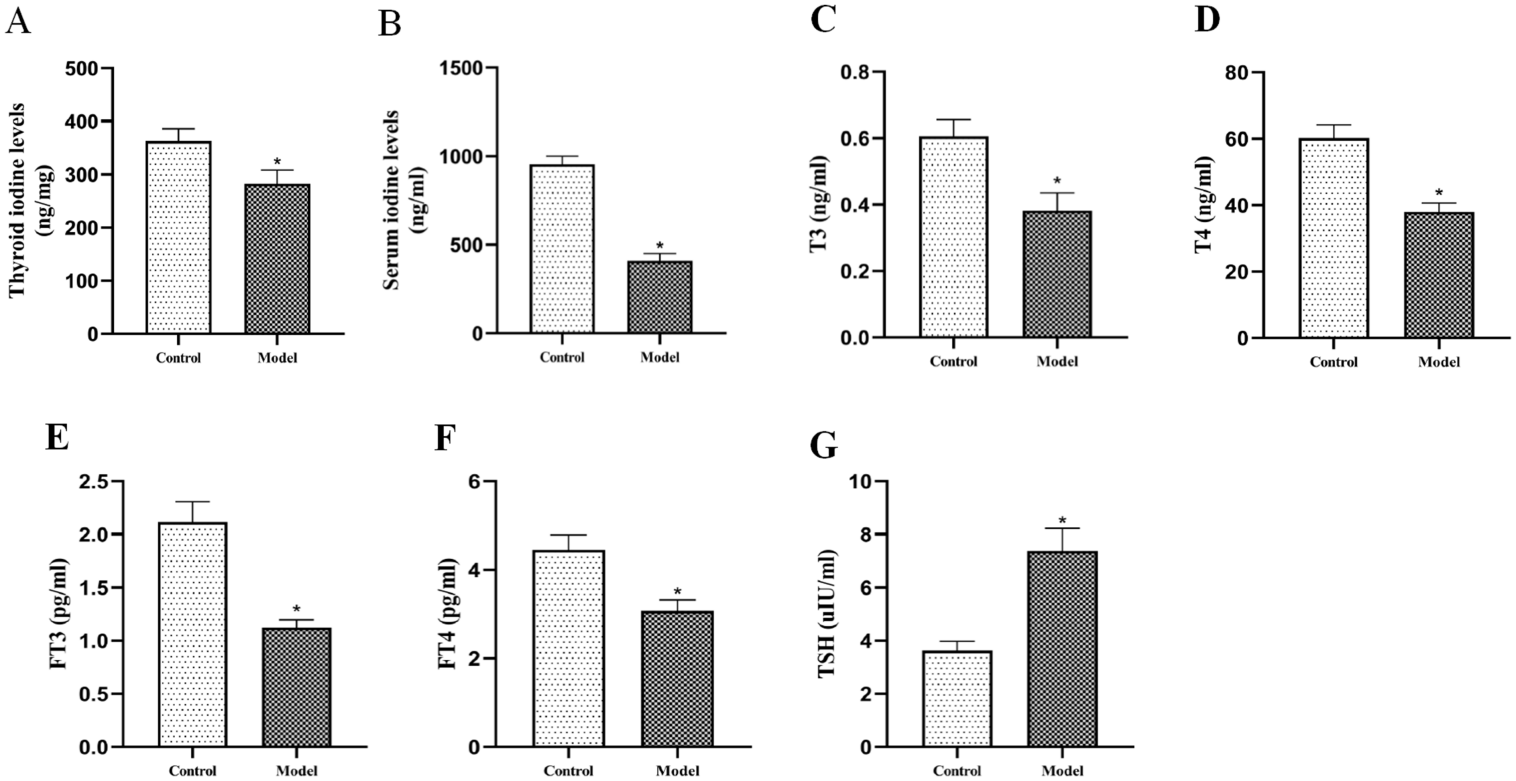
Effects of goiter induction on thyroid iodine metabolism and thyroid function parameters. **(A)** Thyroid iodine levels, **(B)** Serum iodine levels, **(C)** T3, **(D)** T4, **(E)** FT3, **(F)** FT4, and **(G)** TSH levels in control and model groups. *n* = 6, *p < 0.05 compared with the control group.

Serum levels of iodine, T3, T4, FT3, FT4 and thyroid iodine were significantly lower in the model group compared with the control group (Figures 10A–E; *p* < 0.05). In contrast, serum TSH levels were significantly higher in the model group (Figure 10F; *p* < 0.05). These findings demonstrate a pathogenic cascade whereby downregulation of thyroid NIS expression impairs iodine uptake, resulting in insufficient T3/T4 synthesis. This hormonal deficiency triggers pituitary feedback mechanisms that increase TSH secretion. Chronically elevated TSH then stimulates thyroid follicular cell hyperplasia, ultimately promoting goiter formation.

## 4 Discussion

In this study, we employed MR analysis to establish a causal relationship between gut microbiota and goiter. Our findings revealed that 10 gut microbial taxa were causally associated with goiter risk across different taxonomic levels. Specifically, increased abundance of *Ruminococcus gauvreauii* group, Rikenellaceae RC9 gut group, and Ruminococcaceae UCG 003 was associated with a higher risk of goiter, while increased abundance of Cyanobacteria (phylum), Actinobacteria (class), Melainabacteria (class), Gastranaerophilales (order), Lachnospira (genus), *Eubacterium hallii* group (genus), and Bifidobacterium (genus) was associated with a reduced risk of goiter.

To further validate these findings, we established a rat goiter model using PTU and performed 16S rRNA sequencing on fecal samples. The sequencing results demonstrated that goiter induction led to a significant reduction in gut microbial diversity and altered community structure, indicating a disruption in intestinal microbiota homeostasis. Notably, eight gut microbial taxa—Actinobacteriota (phylum), Actinobacteria (class), Bifidobacteriales (order), Bifidobacteriaceae (family), Bifidobacterium (genus), Allobaculum (genus), and Oscillibacter (genus)—showed statistically significant differences between the control and model groups. Of these, Bifidobacterium exhibited significant differences at all taxonomic levels (phylum, class, order, family, and genus).

By integrating MR and 16S rRNA sequencing results, we identified Actinobacteria (class) and Bifidobacterium (genus) as causally linked to goiter. A decrease in their abundance appears to contribute to goiter development, suggesting their potential as key therapeutic targets. Importantly, Bifidobacterium, a member of the class Actinobacteria, emerged as the most critical gut microbiota for goiter treatment.

The gut microbiota plays a pivotal role in maintaining systemic homeostasis, acting as both “endocrine organs” and “metabolic regulators” (Sekirov et al., 2010; Fenneman et al., 2023). Iodine, a micronutrient essential for thyroid hormone synthesis, is primarily absorbed through the gastrointestinal tract and transported to the thyroid gland. Dysregulation of iodine metabolism can impair thyroid hormone synthesis, triggering a negative feedback loop that increases TSH secretion, leading to thyroid hyperplasia and goiter (Sponziello et al., 2010; Hou et al., 2023).

**Figure 11:**
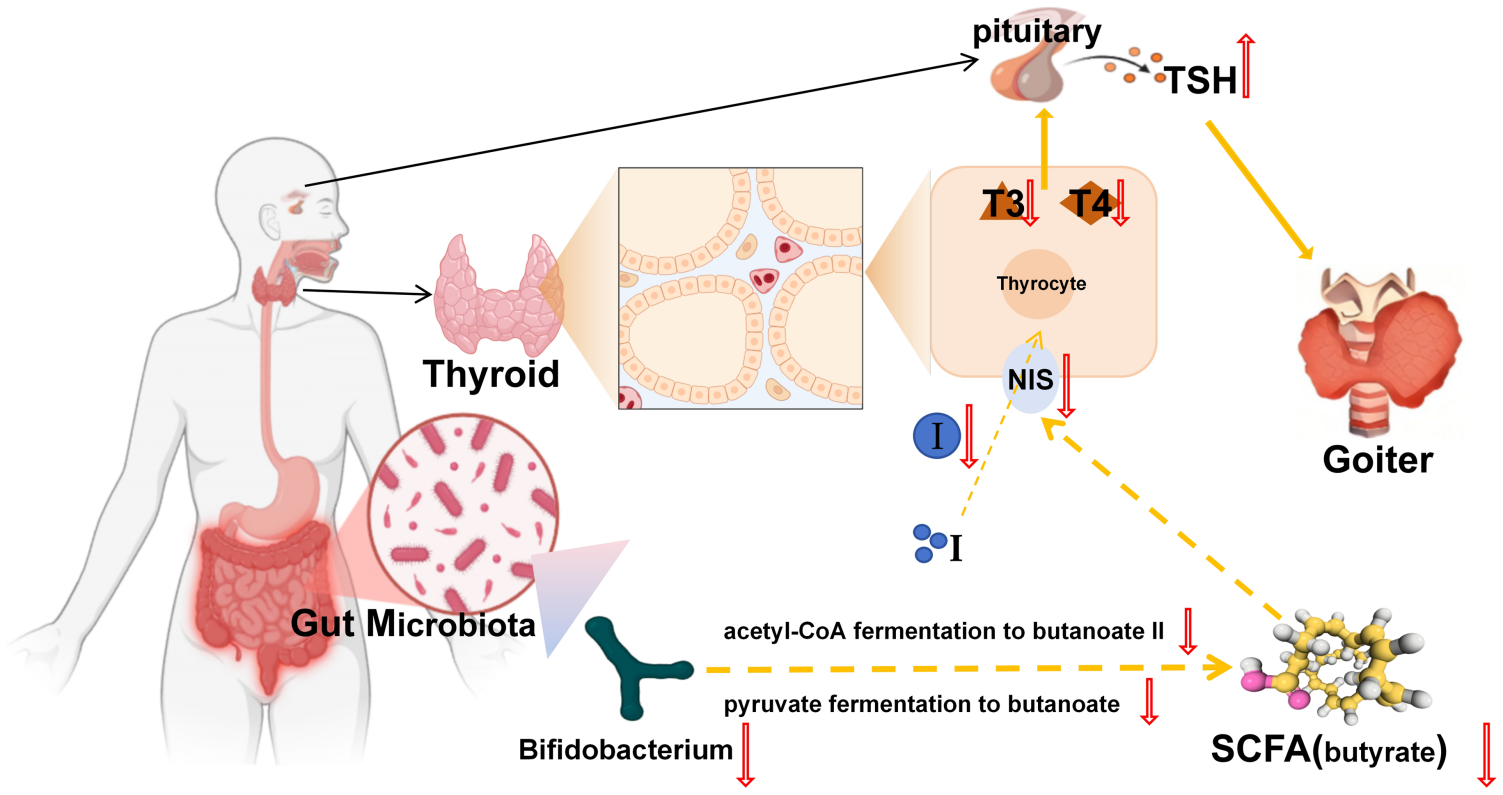
Proposed mechanism of Bifidobacterium depletion-induced goiter pathogenesis. NIS, Sodium-iodide symporter; I, iodine.

The gut microbiota, particularly Bifidobacterium taxa, appears to regulate thyroid function through modulation of iodine metabolism (Lin et al., 2022; Gong et al., 2024). This regulation occurs primarily through microbial metabolites (especially SCFAs) that influence the expression and activity of the NIS, the key transporter responsible for iodine uptake (Li N. et al., 2022; Ashrafian, 2023; Zheng et al., 2023; Lu et al., 2024). Our integrated analyses revealed significant disruptions in SCFA-mediated thyroid regulation in the goiter model. PICRUSt2 metabolic predictions and targeted SCFA quantification consistently demonstrated downregulation of two critical butyrogenic pathways (acetyl-CoA fermentation to butyrate II and pyruvate fermentation to butyrate), accompanied by marked reductions in six SCFAs including butyrate. These metabolic alterations were paralleled by significant decreases in thyroid NIS protein expression, as confirmed by both immunohistochemical staining (reduced NIS-positive cells) and Western blot analysis. These findings align with established evidence that butyrate upregulates NIS expression in thyroid cells (Yang et al., 2019), suggesting a mechanistic link between gut microbiota-derived SCFAs and iodine metabolism. Further supporting this pathway, we observed characteristic thyroid dysfunction markers in the model group: decreased thyroidal and serum iodine levels, reduced circulating T3, T4, FT3, and FT4 concentrations, and compensatory TSH elevation. Collectively, these results substantiate our hypothesis that *Bifidobacterium bifidum* maintains thyroid homeostasis through SCFA-dependent modulation of iodine metabolism, wherein microbial butyrate production appears critical for proper NIS expression and subsequent thyroid hormone synthesis. The experimental results demonstrated a clear pathological cascade: depletion of Bifidobacterium leads to impaired SCFA synthesis, downregulated NIS protein expression, reduced thyroidal iodine uptake, decreased thyroid hormone production, compensatory TSH elevation, and ultimately, goiter development (Figure 11).

Our investigation presents several methodological and conceptual advances in understanding the gut-thyroid axis. Primarily, the application of Mendelian randomization analysis established putative causal relationships between gut microbiota and goiter risk, effectively addressing limitations of conventional observational studies by minimizing residual confounding and reverse causation bias. The systematic evaluation across multiple taxonomic hierarchies (from phylum to genus level) enabled a comprehensive identification of goiter-associated microbial features. To ensure analytical rigor, we implemented stringent instrumental variable selection criteria, including checks for heterogeneity and horizontal pleiotropy, thereby enhancing the validity of our causal inferences. The MR findings were further substantiated through a multimodal validation approach: (1) establishment of a rat goiter model with 16S rRNA sequencing confirmation (*α*/*β*-diversity analysis, differential taxa identification by t-test, and PICRUSt2 metabolic prediction); (2) targeted biochemical assays quantifying SCFA concentrations, iodine metabolism parameters, and thyroid hormone profiles; and (3) histopathological and molecular validation (immunohistochemistry and Western blot analysis of NIS expression). This translational integration of bioinformatic prediction with experimental validation uniquely positions our study to: (i) identify specific gut microbial taxa with causal effects on goiter risk, and (ii) elucidate their mechanistic pathways through SCFA-mediated modulation of iodine metabolism. These findings provide a evidence-based framework for developing microbiota-targeted interventions in goiter management.

While establishing causal gut microbiota-goiter relationships, our study has unresolved mechanistic questions: (i) the precise functional pathways of identified microbial taxa require validation in germ-free models, and (ii) the therapeutic potential of Bifidobacterium-SCFA modulation needs clinical translation through human fecal microbiota transplantation studies. These open questions represent valuable directions for future research.

## 5 Conclusion

Our study provides robust evidence for a causal gut microbiota-goiter relationship through integrated genetic and experimental analyses, identifying *Bifidobacterium bifidum* as a key microbial regulator with therapeutic potential. These findings establish the gut-thyroid axis as a mechanistic pathway in goiter pathogenesis, and highlight microbial modulation as a promising strategy for goiter intervention. Future research should focus on translational validation, including *B. bifidum* monoculture studies and clinical trials evaluating microbiota-targeted therapies.

## Data Availability

The raw 16S rRNA sequencing data have been deposited in the NCBI Sequence Read Archive (SRA) under BioProject accession PRJNA1279838.

## Footnotes

^1^ www.mibiogen.org

^2^ https://www.arb-silva.de
